# Label-Free Detection of the Receptor-Binding Domain of the SARS-CoV-2 Spike Glycoprotein at Physiologically Relevant Concentrations Using Surface-Enhanced Raman Spectroscopy

**DOI:** 10.3390/bios12050300

**Published:** 2022-05-05

**Authors:** Andrey K. Sarychev, Alyona Sukhanova, Andrey V. Ivanov, Igor V. Bykov, Nikita V. Bakholdin, Daria V. Vasina, Vladimir A. Gushchin, Artem P. Tkachuk, Galina Nifontova, Pavel S. Samokhvalov, Alexander Karaulov, Igor Nabiev

**Affiliations:** 1 Institute of Theoretical and Applied Electrodynamics, Russian Academy of Sciences, 125412 Moscow, Russia; sarychev_andrey@yahoo.com (A.K.S.); av.ivanov@physics.msu.ru (A.V.I.); bykov.i.v@yandex.ru (I.V.B.); 2 Laboratoire de Recherche en Nanosciences, LRN-EA4682, Université de Reims Champagne-Ardenne, 51100 Reims, France; alyona.sukhanova@univ-reims.fr (A.S.); galina.nifontova@univ-reims.fr (G.N.); 3 Moscow Power Engineering Institute, National Research University, 111250 Moscow, Russia; baholdin.n@mail.ru; 4 Gamaleya National Research Centre for Epidemiology and Microbiology, Ministry of Health of the Russian Federation, 123098 Moscow, Russia; d.v.vasina@gmail.com (D.V.V.); vladimir.a.gushchin@gamaleya.org (V.A.G.); artem.p.tkachuk@gamaleya.org (A.P.T.); 5 Department of Virology, Biological Faculty, Lomonosov Moscow State University, 119234 Moscow, Russia; 6 Moscow Engineering Physics Institute, National Research Nuclear University MEPhI, 115409 Moscow, Russia; p.samokhvalov@gmail.com; 7 Department of Clinical Immunology and Allergology, Institute of Molecular Medicine, Sechenov First Moscow State Medical University (Sechenov University), 119146 Moscow, Russia; drkaraulov@mail.ru

**Keywords:** SARS-CoV-2, S glycoprotein, RBD, surface-enhanced Raman spectroscopy, Raman spectroscopy, SERS, AFM

## Abstract

Surface-enhanced Raman scattering (SERS) spectroscopy is a surface- or cavity-enhanced variant of Raman scattering spectroscopy that allows the detection of analytes with a sensitivity down to single molecules. This method involves the use of SERS-active surfaces or cavities capable of concentrating incident radiation into small mode volumes containing the analyte. Here, we have engineered an ultranarrow metal–dielectric nano-cavity out of a film of the receptor-binding domain (RBD) of SARS-CoV-2 spike (S) glycoprotein and a silver surface, held together by interaction between reduced protein sulfhydryl groups and silver. The concentration of light in this nano-cavity allows the label-free recording of the characteristic Raman spectra of protein samples smaller than 1 pg. This is sufficient for the ultrasensitive detection of viral protein antigens at physiologically relevant levels. Moreover, the protein SERS signal can be increased by several orders of magnitude by coating the RBD film with a nanometer-thick silver shell, thereby raising the cavity Q-factor. This ensures a sub-femtogram sensitivity of the viral antigen detection. A simple theoretical model explaining the observed additional enhancement of the SERS signal from the silver-coated protein is proposed. Our study is the first to obtain the characteristic Raman and SERS spectra of the RBD of S glycoprotein, the key SARS-CoV-2 viral antigen, directly, without the use of Raman-reporter molecules. Thus, our approach allows label-free recording of the characteristic spectra of viral antigens at concentrations orders of magnitude lower than those required for detecting the whole virus in biological media. This makes it possible to develop a high-performance optical detection method and conformational analysis of the pathogen and its variants.

## 1. Introduction

Three coronaviruses have crossed the species barrier to cause deadly pneumonia in humans since the turn of the 21st century: severe acute respiratory syndrome coronavirus (SARS-CoV) [1,2], Middle-East respiratory syndrome coronavirus (MERS-CoV) [3], and SARS-CoV-2 [4,5]. The SARS-CoV infection emerged in the Guangdong province of China in 2002 and spread to five continents through air travel routes, infecting 8098 people and causing 774 deaths. In 2012, MERS-CoV caused an epidemic in the Arabian Peninsula, where it still remains a major public health concern, and spread to 27 countries, infecting a total of 2494 individuals and claiming 858 lives. Finally, a previously unknown coronavirus, named SARS-CoV-2, was discovered in December 2019 in Wuhan, the Hubei province of China. SARS-CoV-2 is responsible for the ongoing outbreak of atypical pneumonia (COVID-2019) that has affected nearly 511 million people and killed over 6.2 million in more than 220 countries worldwide as of 27 April 2022 (www.worldometers.info/coronavirus/, accessed on 27 April 2022). Recurrent spillovers of coronaviruses in humans, together with findings of numerous coronaviruses, including SARS-like ones (SARSr-CoV) in bats, suggest that future zoonotic transmission events are highly probable [6].

The global world pandemic situation related to COVID-19 could have been prevented if large-scale, quick diagnosis of active infection cases had been possible. This further confirms that more efficient methods for detecting viral infections are to be developed. The existing immune [7] and molecular [8] assays, including ELISA [9] and PCR [10], are sufficiently sensitive for the identification of coronaviruses but require prior knowledge of the strains. Deep sequencing techniques [11], which can detect mutations in virus genomes and capture information on the virus’s genetic diversity if the sequence coverage is sufficient, are powerful virus surveillance tools. However, because of low virus titers in most clinical samples, sequence reads are usually dominated by host rather than viral genetic material. Furthermore, the processing of samples is time-consuming, requires expensive reagents, and involves high technical expertise. The development of a highly sensitive and specific spectral method for the quick detection of coronaviruses, particularly SARS-CoV-2 strains, could reduce both the time and cost of the detection, ensure earlier diagnosis, and save many lives.

**Figure 1:**
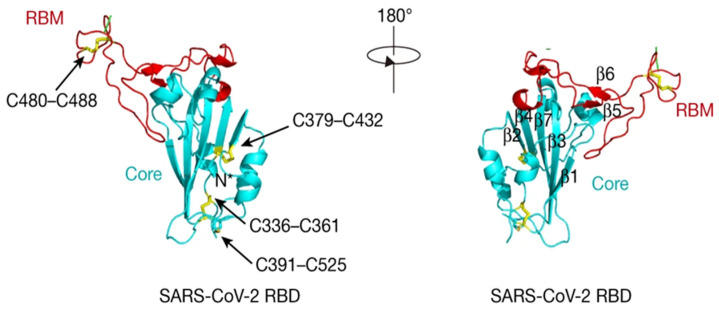
The three-dimensional structure of the receptor-binding domain (RBD) of the SARS-CoV-2 spike (S) glycoprotein. The RBD is shown in cyan, and the receptor-binding motif (RBM) of this protein, which interacts with the cell receptor, is shown in red. The SARS-CoV-2 RBD disulfide bonds are shown in yellow and marked with arrows. Adapted with permission from ref. [13]. Copyright 2020 Nature.

The selection of the optimal spectral detection object in the virus, with an optical signal not only intense, but also varying in different strains, is the key task in developing a highly sensitive and specific quick diagnostic method. Coronavirus entry into host cells is mediated by cell interaction with the spike (S) glycoprotein, which protrudes outside the virus envelope [12] and includes a receptor-binding domain (RBD, see Supplementary Tables S1 and S2). The RBD ensures the recognition of the cell receptor and the fusion of the virus and cell membranes, resulting in the virus’s entry into the cell (Figure 1). Because the coronavirus S glycoprotein (including the RBD) is both exposed on the surface and crucial for the entry into cells, it is the main target of neutralizing antibodies and the focus of drug and vaccine development. At the same time, the possible structural/morphological differences between the S glycoproteins of SARS-CoV-2 and other coronaviruses, as well as between S glycoproteins of different viral strains, can be considered as a basis for differential diagnosis of viral strains and variants.

Optical sensing technologies have been widely recognized to hold the key to the development of quick, high-throughput, easy-to-use, point-of-care diagnostics. A number of surface-enhanced Raman scattering (SERS) spectra of biological molecules, including amino acids, peptides, proteins (water-soluble, membrane, and glycosylated ones), nucleotides, oligonucleotides, and double-stranded and single-stranded DNAs, as well as SERS spectra of viruses, bacteria, and drug compounds [14,15,16], have been published since the early 1980s. It has been demonstrated that the SERS spectra of glycoproteins can be recorded with a sensitivity down to their amounts in single virions [17,18,19]. In addition, equipment and technologies for the molecular analysis of substances based on tip-enhanced Raman spectroscopy (TERS) have recently been developed [20,21]. All this makes the ambitious task of the specific optical detection of the SARS-CoV-2 S glycoprotein within seconds a reality. However, to date, only a handful of studies have reported the detection of SARS-CoV-2 viral particles or its protein antigens at physiologically relevant concentrations. Two optical sensing approaches have been used in this field, one based on surface plasmon resonance (SPR) and the other based on SERS; see, e.g., [22,23,24,25,26,27,28] and Supporting Materials. The most sensitive SERS detection of SARS-CoV-2 and its spike and nucleocapsid proteins known to date employed silver–copper microstars over 400 nm in size, i.e., of the order of the virion size [25]. Importantly, all the studies on SERS detection of SARS-CoV-2 viral particles or its protein antigens published to date are indirect. They record the SERS spectra of the so-called SERS nanotags, labeled with low-molecular-weight Raman reporters, or those of the receptors or aptamers binding S glycoprotein, rather than the SERS spectra of the viral antigens themselves [23,24,25,26,27,28]. The use of such indirect approaches makes it impossible to study the structure of protein antigens and their differences in different variants of the virus.

To the best of our knowledge, our study is the first to obtain typical Raman and SERS spectra characteristics of the RBD of S glycoprotein, the key SARS-CoV-2 viral antigen. An approach has been developed that allows the formation of metal–dielectric microcavities via the interaction of the reduced thiol groups of the RBD with the SERS-active silver surface. The new approach makes it possible to enhance the very short-range Raman signals and obtain SERS spectra of viral protein antigens at concentrations sufficiently low for their detection at physiologically relevant (sub-picogram) levels. Moreover, we have demonstrated that the Q-factor of the metal–dielectric microcavity can be increased by coating the dielectric RBD film with a silver shell several nanometers in thickness, which leads to an order(s)-of-magnitude enhancement of the RBD SERS signal. A simple theoretical model qualitatively describing the observed additional enhancement of the SERS signal from the protein coated with a thin silver film is proposed.

The principal difference of our approach from the previous ones is the possibility of label-free recording of the characteristic protein Raman spectra of the SARS-CoV-2 antigen without using principal component analysis and at concentrations orders of magnitude lower than those required for the detection of the whole virus in biological media. The obtained results pave the way to the development of a reliable high-performance optical method not only for the detection of the pathogen, but also for conformational analysis of its variants.

## 2. Materials and Methods

### 2.1. SARS-CoV-2 RBD

The recombinant RBD is an Arg319–Phe541 fragment of the S1 glycoprotein of SARS-CoV-2 fused to a His6 tag at the C terminus (Figure 1) [13]. The RBD sequence and physico-chemical properties are presented in the Supplementary Tables S1 and S2. The recombinant RBD was expressed in mammalian cells and purified using metal affinity chromatography (RBD lot no. 8COV1, HyTest, Moscow, Russia). The protein molecular weight was calculated to be 25,921 Da (including the histidine residues); the purity of the protein preparation was more than 95%.

About 1 mg of the protein was dissolved in 140 μL of ultrapure water (MilliQ, 18.2 MΩ×cm), additionally filtered through 0.22-μm Millipore filters (Merck, Cork, Ireland) and transferred into a 0.01 M phosphate buffer solution (pH 7.2), using gel-filtration chromatography by means of PD MiniTrap G-25 columns (GE Healthcare, Buckinghamshire, UK). The protein concentration in the obtained eluates was determined using the Bradford method by means of a Nanodrop 2000 instrument (Thermo Fisher Scientific, Wilmington, NC, USA). Quality control of the protein samples in the phosphate buffer solution was performed by electrophoresis in polyacrylamide gel according to Laemmli, in the presence of sodium dodecyl sulfate under reducing and nonreducing conditions. Detection was performed using a ChemiDoc XRS+ instrument (Bio-Rad, Hercules, CA, USA).

Disulfide bonds in the RBD structure (Table S1), indicated by arrows in Figure 1, were reduced using tris(2-carboxyethyl)phosphine hydrochloride (TCEP) (Thermo Fisher Scientific, Rockford, IL, USA); the sample was purified from excess TCEP and transferred into a 0.01 M phosphate buffer solution (pH 6.0) using MiniTrap G-25 columns (GE Healthcare, Buckinghamshire, UK). As a result, a sample of purified RBD protein with reduced disulfide bonds at a concentration of 1.2 mg/mL was obtained.

It should be noted that an approach employing the structure of protein disulfide bonds for its label-free detection using SERS was demonstrated for human insulin [29] and is discussed later in [30]; the reference database of Raman spectra signatures of biological molecules which may also be used for the SERS spectra interpretation is presented in [31].

### 2.2. Preparation and Characterization of SERS-Active Substrates

Cleaned 26 × 10 mm Menzel Gläser slides (Thermo Fisher Scientific, Waltham, MA, USA) were placed into a vacuum chamber at a pressure of 10^−2^ Torr and treated with a glow discharge at a current of 0.3 A and a power of 1.5 kW for 30 min, after which the pressure was lowered to 2 × 10^−5^ Torr. Silver was sputtered from a molybdenum crucible for 1.5 min using electron-beam heating at a current of 40 mA and a voltage of 8 kV. The thickness of the sputtered silver layer was estimated by the transmission in the optical channel of the test sample at a wavelength of 600 nm to a value of T ≈ 0%, which corresponded to a silver film thickness of 90–100 nm. The parameters of the sputtering procedure were adjusted so as to form silver surface nano-roughness, favorable for the excitation of localized surface plasmons. After that, the RBD sample was applied onto the silver surface as described below.

If additional silver sputtering on the protein sample was required, the slides were placed into a vacuum chamber at a pressure of 2 × 10^−5^ Torr without glow discharge treatment. Silver was sputtered from a molybdenum crucible for 0.5 min using electron-beam gun heating at a current of 40 mA and a voltage of 8 kV. The thickness of the sputtered silver was estimated from the transmission in the optical channel of the test sample at a wavelength of 600 nm to the value of T = 50%, which corresponded to a silver thickness of 10 nm.

**Figure 2:**
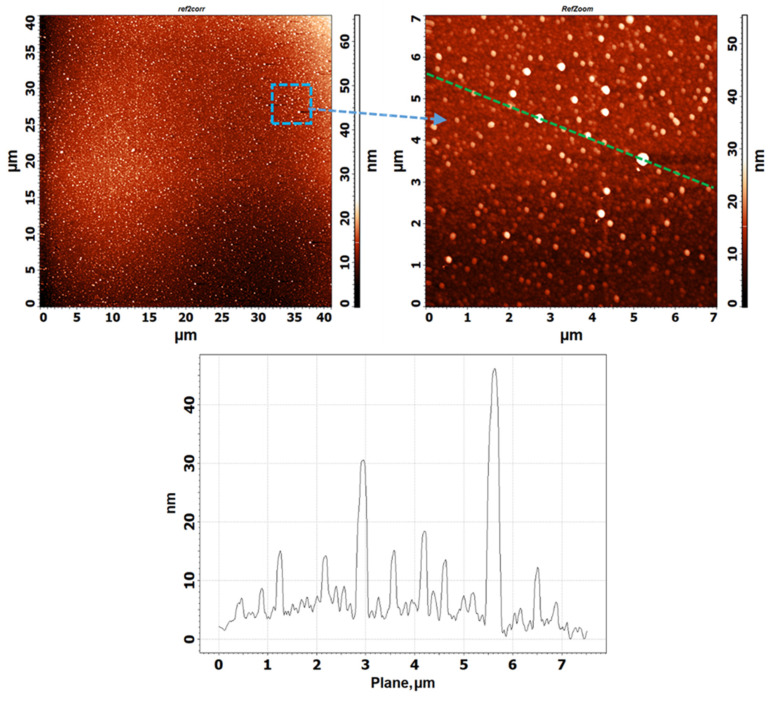
AFM images of the metal surface of surface-enhanced Raman scattering (SERS)-active substrates without protein samples applied. The left panel shows a 40 × 40 μm area; the right panel shows the image obtained in the area marked with a blue square in the left panel (7 × 7 μm); the bottom panel shows the cross-section along the green line in the right panel.

After the formation of SERS-active substrates, they were examined by means of optical microscopy and atomic force microscopy (AFM) in order to detect defect-free areas for the application of protein samples. The studies were performed by means of a Probe Optical 3D Correlation Microscopy System, a unique research setup using VIT_P/IR semi-contact “top-visual” AFM probes (TipsNano, Tallinn, Estonia), at a resolution of 512 × 512 points per scan and a scan rate of 0.8 Hz. As a result, suitable 4 × 4 mm areas were selected and marked for the positioning procedure before exposure in the protein solution. Figure 2 shows typical AFM images of the metal surface of SERS-active silver substrates. It can be seen that rough metal deposits were formed with lateral sizes of about 0.5 μm and average heights within 10–30 nm. The silver bumps and pits operate as open plasmon resonators and generate a large local electric field excited by the impinged electromagnetic wave, with the EM field reaching its maxima in metal depressions [32,33,34]. These data were subsequently used for identifying and analyzing the protein samples applied onto the SERS-active substrates.

### 2.3. Application of the Solutions Containing the RBD of the SARS-CoV-2 S Glycoprotein onto the Surface of Glass or SERS-Active Substrates

**Figure 3:**
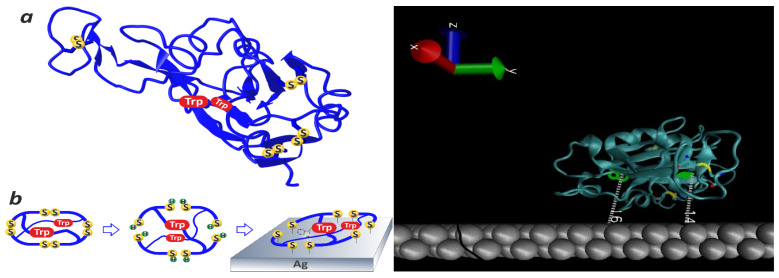
Left: A sketch illustrating the method for fabricating a metal–dielectric microcavity consisting of a dielectric protein globule of the RBD and the metal surface of the SERS-active substrate. The reduction of all four disulfide bonds of the RBD protein results in the formation of an additional eight free sulfhydryl groups capable of forming strong chemical bonds with silver atoms. The unfolding of the protein globule as a result of disulfide bond reduction leads to spatial approximation between the tryptophan (Trp) residues and the surface of the SERS-active substrate and the dominance of Trp in the SERS spectrum (see the data presented in Section 3.1 below). Right: A sketch of the structure of the native RBD of the SARS-CoV-2 S glycoprotein applied onto the silver surface of the SERS-active substrate. The distances between the surface of the SERS-active substrate and two Trp residues of the RBD amino acid sequence located deep in the protein globule are indicated. The data were calculated from the atomic coordinates of the three-dimensional structure of the RBD protein reported in [13].

**Scheme 1:**
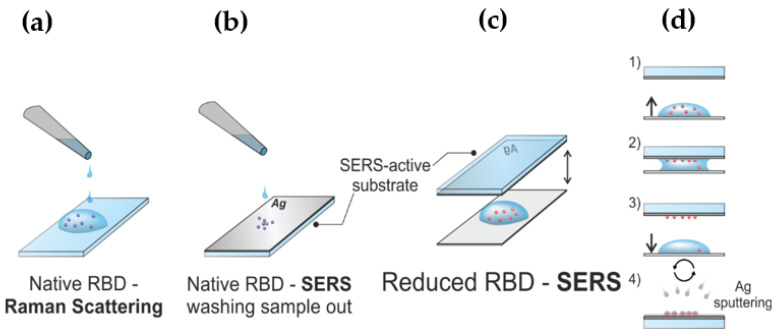
A sketch of the procedure of sample deposition and treatment for recording Raman and SERS spectra. (**a**) For recording the conventional Raman spectrum of the native RBD, the protein solution was applied dropwise onto a glass slide and dried. The Raman spectra were recorded from a sample volume of ~10^9^ nm^3^, which contained about 1.3 pg of protein. (**b**) For recording the SERS spectra of the native RBD, the protein solution was applied dropwise onto defect-free areas of the SERS-active metal film. After incubation, the substrates were washed with water. The SERS spectra were recorded from a sample volume of less than 2.2 × 10^6^ nm^3^, which contained about 2.7 fg of protein. (**c**) For recording the SERS spectra of the reduced RBD, the SERS-active substrate was fixed, with the SERS-active silver side down. A drop of protein solution was applied onto the hydrophobic surface of the Parafilm film mounted on a slide. (**d**) Then, the slide was positioned in such a way that a defect-free region of the SERS-active silver substrate was strictly above the RBD drop (**d**1); the vertical (from bottom upwards) approach of the slide to the SERS-active substrate resulted in the drop spreading over the parafilm upon touching the SERS-active substrate (**d**2). After incubation, the SERS-active substrate was removed from the bench (**d**3) and washed intensely with a stream of water. The SERS spectra were recorded from a single protein aggregate shaped as a disk 300 nm in radius and 4 nm in height, which contained about 1 fg of protein. Application of an additional, optically transparent layer of silver onto the protein (**d**4) resulted in further order(s)-of-magnitude enhancement of the RBD SERS signal.

The native RBD contains one free thiol group and four disulfide bonds [21], whereas the RBD with four reduced disulfide bonds contains nine free thiol groups (Table S1). These free thiol groups, which are capable of forming strong S–Ag chemical bonds with silver surfaces (Figure 3), were applied onto the surface of the SERS-active substrates by vertical exposure (Scheme 1). The experimental bench for sample application consisted of a substrate mounting system and a system for the lateral positioning and the vertical feeding of a drop of protein solution. The substrate was fixed, with the silver SERS-active side down, parallel to the plane of the protein solution drop application. A drop of protein solution (2 μL) was applied onto the hydrophobic cleaned surface of the Bemis™ Parafilm™ M film (Thermo Fisher Scientific, Waltham, MA, USA) mounted on a slide (Menzel Gläser, Thermo Fisher Scientific, Waltham, MA, USA). Then, the Parafilm™ with the solution drop on it was positioned in such a way that a defect-free region of the SERS-active silver substrate was strictly above it. The vertical (from bottom upwards) approach of the Parafilm™ carrying the drop of the protein solution resulted in drop spreading over the Parafilm™ upon touching the SERS-active substrate (Scheme 1). After 5 min of incubation, the SERS-active silver substrate was removed from the bench and washed intensely with a stream of MiliQ water for several minutes. The resultant samples were placed into a desiccator equipped with an air evacuation system for 24 h, after which they were used for measurements.

The described approach excludes the gravitational deposition of the sample onto the surface of the SERS-active substrate and ensures the formation of a uniform protein molecular layer firmly bound to the substrate surface due to Ag–S chemical bonds. It should be noted that although the aliphatic index of the RBD protein (the relative volume occupied by the aliphatic side chains of its amino acid residues) is 71.2% (Table S2), a significant hydrophobic component of this protein (28.8%) [13] appears to be sufficient for the formation of individual protein aggregates on the surface, which are seen in AFM images.

**Figure 4:**
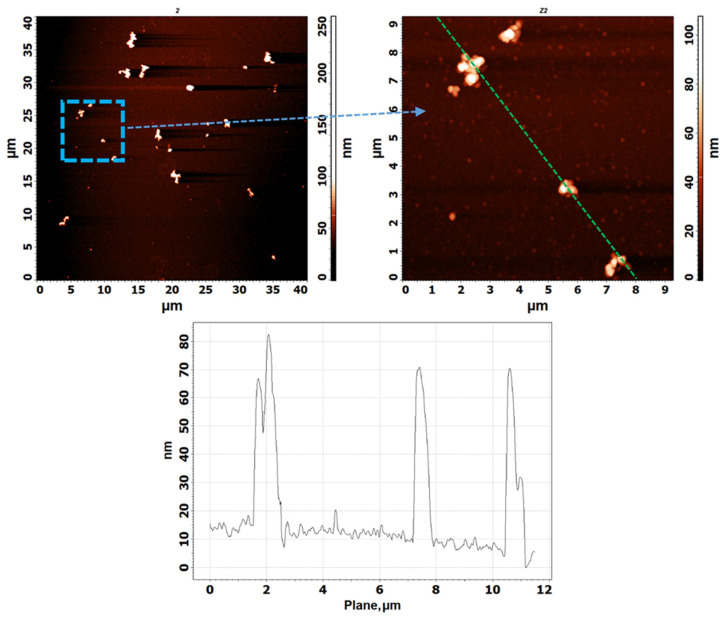
AFM images of the silver surface of the SERS-active substrate with a sample of the RBD of the SARS-CoV-2 S glycoprotein with reduced disulfide bonds applied onto it. The left panel shows a 40 × 40 μm area; the right panel shows the image obtained in the area marked with a blue square in the left panel (9 × 9 μm); the bottom panel shows the cross-section along the green line in the right panel.

Figure 4 shows a typical AFM image of the metal surface of a silver SERS-active substrate, with the RBD sample with reduced disulfide bonds applied onto it as described above (Scheme 1, Figure 3). The RBD-covered areas contain distinct formations with standard lateral dimensions of the order of 0.6 μm and a height of about 50 nm, as seen from Figure 4. It is evident that the areas of protein application are clearly different from the original metal surface shown in Figure 2. Furthermore, there is a distinct internal structuring of these formations, in which they significantly differ from the inhomogeneities of silver. This suggests that they are bulk aggregates of the RBD protein. Accordingly, the main signs of the presence of silver SERS-active areas in the optical image of the studied samples are well-distinguishable inhomogeneities (about 0.6 µm) against the background within the dirt-free areas of the metal film.

Importantly, the described vertical (from the bottom upwards) method of applying the reduced RBD samples onto the surface of our SERS-active substrates (Scheme 1, Figure 3) is inapplicable to the native RBD protein, where the disulfide bonds are not reduced, and hence, the additional free thiol groups are absent. Therefore, the samples of the native RBD protein were completely washed off the surface of the SERS-active substrate, and it was impossible to detect any traces of the protein in the AFM images of these substrates or SERS signals from them. Therefore, to study the native RBD of the SARS-CoV-2 S glycoprotein, the protein solution was applied in drops (2 μL) directly onto the defect-free areas of the SERS-active metal film (Scheme 1), and the film was placed into a desiccator, which was sealed and evacuated immediately after the application of the drops. After incubation in the desiccator for 24 h, the substrates were washed twice for 2 min in water (MiliQ) and placed into the desiccator for another 24 h.

To record the conventional Raman spectra of RBD, as a negative control, the protein solution was applied onto the surface of a clean Menzel Gläser slide (Thermo Fisher Scientific, Waltham, MA, USA), without metal sputtered onto it, and the procedure described above was reproduced.

### 2.4. Mapping the RBD Protein Samples on the SERS-Active Substrate Surface

Spectral measurements were performed using a WITec 500 Alpha Raman spectrometer based on a confocal microscope with a mapping option; the mapping area was 25 × 25 μm. The combination of a spectrometer with an optical microscope made it possible to focus the laser beam and record the spectra from individual protein globules or even individual parts of the globules. The step between the coordinates in the optical image, as well as in the Raman spectrum map, was approximately 1 µm. A laser with a wavelength of 785 nm, a power of 2–3 mW, and a 50× objective lens was used for mapping; the signal accumulation time was 2–5 s.

### 2.5. Raman and SERS Spectroscopy Analysis in a Metal–Dielectric Microcavity

In all the spectral experiments, the position of the sample was fixed, the accumulation time was 1 s, and the wavelength of the excitation laser radiation was 785 nm, at a power of 5.76 mW. The spectra shown in the figures are the results of averaging over 64 spectra.

## 3. Results and Discussion

**Figure 5:**
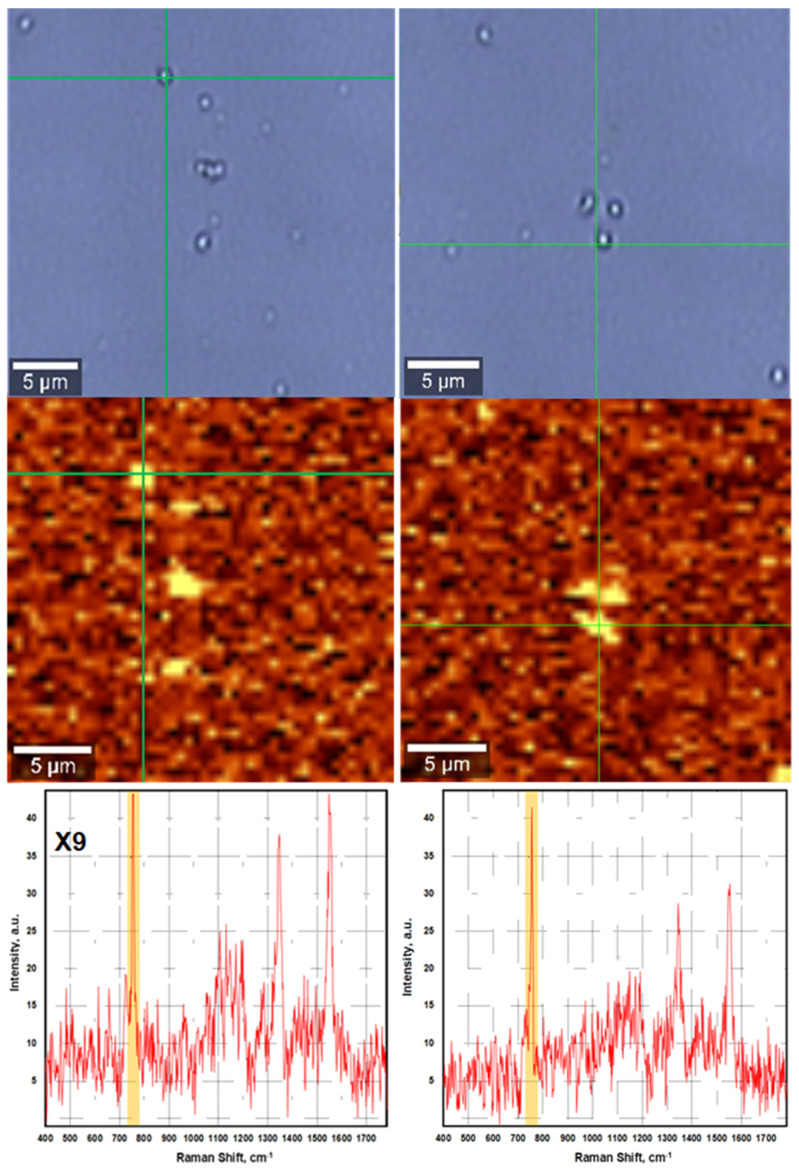
SERS-mapping of a sample of the RBD protein with reduced disulfide bonds. Finding and SERS-mapping a sample of the RBD protein with reduced disulfide bonds applied onto the metal surface of the SERS-active substrate (the left column) and with an additional silver layer applied above (the right column). The upper panels show images in the wide-field upright optical microscopy mode. The middle panels show the confocal SERS-mapping in the characteristic band of the tryptophan line at about 755 cm^−1^ for subsequent recording of the SERS spectrum at the point indicated by the green cross (the lower panels). The scaling factor for the spectrum in the left lower panel is 9.

The SERS mapping of the samples of RBD protein with reduced disulfide bonds on the surface of the SERS-active substrate (Figure 5) demonstrated a distinct and reproducible Raman signal from individual protein aggregates ~0.6 µm in diameter, with the most intense signals observed at the boundaries of the aggregates. Indeed, direct contact of the analyte with the plasmonic substrate is important for their short-range interaction and for amplification of the Raman signal [16].

The Raman signal from the RBD protein on the metal surface resulted from its interaction with plasmons excited in a depression of the metal film. In other words, the SERS signal of the RBD protein was observed due to the excitation of the plasmon resonance and the enhancement of the electric field in the open resonators formed by depressions in the silver film that were filled with the dielectric RBD protein. It is safe to suppose that the Raman signals were mainly generated in the regions of the RBD that had chemical bonds with the silver depression. Therefore, the signal had maxima at the boundaries of the globules where the Raman signal was not screened by the RBD globule. The parameters of the deposition of the silver substrate were tuned in such a way as to increase the amplitude of the random plasmon field generated by the impinging light [33], although the substrate should be further optimized.

### 3.1. The Sensitivities of Raman and SERS Detection of the SARS-CoV-2 RBD

**Figure 6:**
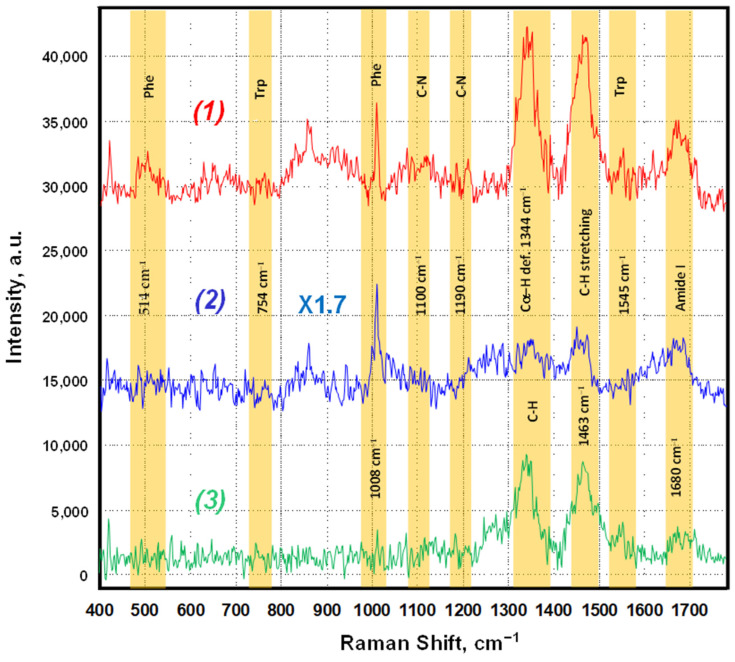
Raman and SERS spectra of the native RBD of the SARS-CoV-2 S glycoprotein. (1) An unenhanced Raman spectrum obtained from the native SARS-CoV-2 RBD from a dried drop on a glass slide. The Raman signal is collected from a focus spot with a volume estimated at 10^9^ nm^3^, which contains about 1.3 pg of RBD. (2) A SERS spectrum obtained from about 2.7 fg of the sample of the native SARS-CoV-2 RBD in a single aggregate on the silver surface of the SERS-active substrate. The scaling factor for the integrated intensity is 1.7. (3) A “Raman minus SERS” difference spectrum demonstrating the SERS effect expressed in the variation of the relative intensities of some Raman bands on the SERS-active substrate.

For conventional Raman spectrum recording, the RBD solution was applied dropwise onto a glass slide and dried. As is discussed in the Section 2 and shown in Table S2, although the aliphatic index of the RBD is 71.2%, the substantial hydrophobic component of this protein (28.8%) appears to be sufficient for the formation of protein aggregates with sizes of several hundred micrometers in all dimensions on the silver surface (data not shown). Hence, it can be assumed that the spectra were recorded from a sample volume of ~10^9^ nm^3^, which corresponded to the excitation volume of the spectrometer. Considering that the volume of the RBD molecule 4 nm in diameter [13] is ~34 nm^3^, the conventional Raman spectra of the native RBD (Figure 6, spectrum 1) were recorded from about 1.3 pg of the sample.

The RBD Raman spectrum recorded for this sample (Figure 6, spectrum 1) is typical of a water-soluble protein [31]. This spectrum is dominated by peaks at 1463 cm^−1^, attributed to C–H stretching, and at 1344 cm^−1^, attributed to C–H deformation; it also clearly displays the bands of the tyrosine (at about 858 cm^−1^), phenylalanine (about 1008 cm^−1^), amide III (about 1265 cm^−1^), and amide I (about 1670 cm^−1^) vibrations. At the same time, no sharp bands at 761, 1330, or 1561 cm^−1^, which are characteristic of tryptophan residues, appear in the Raman spectrum of RBD, which is due to the relatively low tryptophan content of the RBD protein: two tryptophan residues versus 14 phenylalanine residues in the total RBD sequence of 223 amino acid residues (see Table S2 for the RBD amino acid composition).

The SERS spectrum of the native RBD (Figure 6, spectrum 2) was obtained from a single protein aggregate. The spectra were recorded from the location of the drop of the native protein solution, which was washed off before recording the spectra. The shape of the protein aggregates can be approximately estimated from Figure 4 as segments of spheres with a diameter of 600 nm and a height of 50 nm. The volumes of these segments can be routinely estimated to be less than 2.2 × 10^6^ nm^3^. Therefore, the SERS signal of the native RBD was recorded from 6.4 × 10^4^ molecules of RBD, which corresponded to 2.7 fg of the RBD sample.

Comparison of the Raman and SERS spectra of the native RBD of SARS-CoV-2 S glycoprotein (Figure 6, spectra 1 and 2) shows that the amplitude of the Stokes lines, measured from the base line, are approximately the same for both spectra. However, the amount of RBD in these samples differs considerably. The Raman spectrum of the native RBD (spectrum 1) was obtained from ~1 pg of the protein, whereas the SERS spectrum of the native RBD (spectrum 2) was obtained from as little as 1 fg of the protein. Therefore, given the close values of the scaling factors of spectra 1 and 2, the enhancement factor for the SERS spectrum of the native RBD can be estimated as *G*_1_~10^3^. The shape of the SERS spectrum of the native RBD is very similar to the shape of the Raman spectrum of this protein (compare spectra 1 and 2 in Figure 6). In order to further confirm the SERS spectral effect related to the interaction of the protein molecules with the SERS-active surface, we have analyzed the difference spectrum between the Raman and the SERS spectra (Figure 6, spectra 1 and 2) for the native RBD deposited on glass and on the SERS-active surface, respectively (Figure 6, spectrum 3). One can see that the Raman and SERS spectra of the native RBD match well in the region of 400–1200 cm^−1^, yielding a practically flat baseline after subtraction. This region includes characteristic bands of the aromatic amino acids Phe, Trp, and Tyr and some stretching vibrations of the protein backbone. Beyond this region, the difference spectrum shows clear differences between the Raman and the SERS signals of the native RBD protein in the regions of C–H stretching, C–H deformation, and amide I vibrations. This confirms the existence of specific interactions of RBD with the SERS-active surface, which may have determined the so-called “chemical component” of the mechanism of the observed 10^3^-fold enhancement of the RBD Raman signal [15,16].

**Figure 7:**
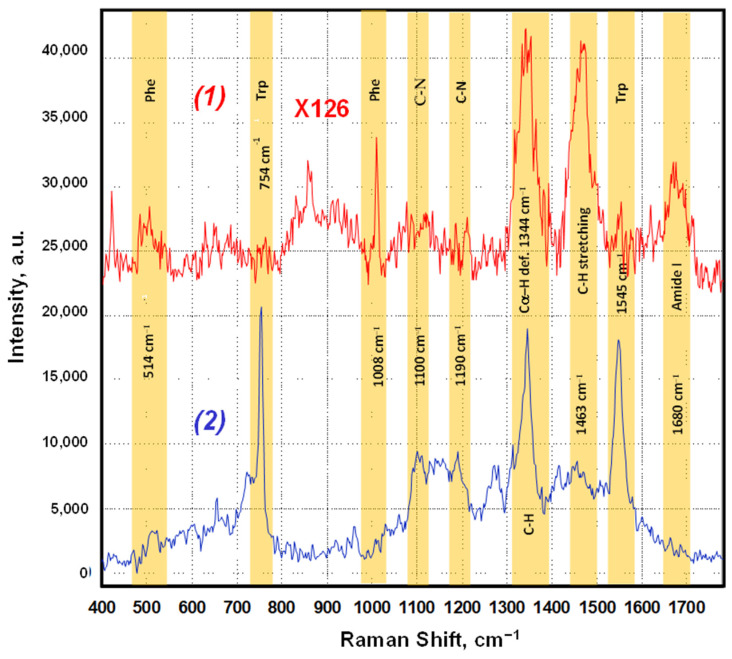
SERS spectral features of the SARS-CoV-2 RBD with reduced disulfide bonds. (1) An unenhanced Raman spectrum obtained from the native SARS-CoV-2 RBD from a dried drop on a glass slide, about 1.3 pg of RBD. The scaling factor for the integrated intensity is 126. (2) A SERS spectrum obtained from about 1 fg of the sample of SARS-CoV-2 RBD with reactive thiol groups (reduced disulfide bonds) on the silver surface of the SERS-active substrate demonstrating the dominance of the Trp amino-acid residue signals.

In the case of the RBD with reduced S–S bonds (which provided an additional eight free SH groups with a high affinity for the silver surface), the Raman signal was obtained from the area of a single protein aggregate that was in direct contact with the silver surface. Such an aggregate can be approximately described as a semi-ellipsoid with a radius of *a* ≈ 200 nm and a height of *h* ≈ 40 nm, whose effective volume can be estimated as *V*_ef_ = 2π*a*^2^*b*/3~3 × 10^6^ nm^3^. Applying the same volumetric approach to this case, we can estimate the quantity of the reduced RBD determining the SERS signal at about 3 fg. Considering that the scaling factor for the unenhanced Raman spectrum compared to the SERS spectrum of the reduced RBD is 126 (Figure 7), the SERS enhancement factor for the RBD with reduced disulfide bonds can be estimated to be at least *G*_2_~126 × *G*_1_~10^5^, i.e., at least two orders of magnitude larger than *G*_1_.

Importantly, when the RBD protein with four reduced disulfide bonds (Table S1) was deposited on the SERS-active substrate, the shape of its SERS spectrum was completely different from the Raman spectra of the native RBD (Figure 7). The spectrum of the reduced RBD is dominated by previously absent bands of the aromatic amino acid tryptophan (Trp) at about 755, 1340, and 1545 cm^−1^. The characteristic signals of Phe, Tyr, and amide I completely disappeared, while the amide III vibration at about 1265 cm^−1^ and the C–H deformation vibration at about 1455 cm^−1^ were strongly suppressed (Figure 7, spectrum 2). Such a significant change in the Raman spectrum, along with a significant enhancement by the SERS-active substrate, indicates the predominant role of the directional binding of the reduced RBD to the silver surface through the thiol groups that were formed by the reduction of the RBD disulfide bonds. A sketch demonstrating this mode of interaction of the reduced RBD with the surface of the SERS-active substrate is shown in Figure 3.

As noted above, the increase in the signal enhancement factor for the reduced RBD by two orders of magnitude compared to the native RBD can be understood if we assume that the *G*_2_ enhancement factor is mainly determined by the direct interaction of the RBD fragments with the silver substrate via thiol binding. Therefore, the amount of RBD protein that effectively generates the SERS signal can be assumed to correspond to one molecular monolayer, i.e., 1 fg of RBD. This assumption is further confirmed by the close similarity between the ordinary Raman spectrum of the native RBD and its SERS spectrum (Figure 6, spectra 1 and 2). The slight difference, clearly shown in spectrum 3 (Figure 6), can be explained by the interaction of the native RBD molecules with a highly inhomogeneous plasmon field excited in the SERS silver substrate [32,33,34]. On the other hand, the SERS signal of the reduced RBD (Figure 7, spectrum 2) is not only a hundred times more intense than that of the native RBD, but also has a rather different shape. This could be due to the formation of thiol bonds between the RBD and the SERS substrate, which indirectly modulates the intensity of the Stocks lines from the vibrations that are not physically associated with these bonds. This phenomenon, sometimes called “chemical enhancement”, is still widely discussed [35,36]. However, we have observed direct transition from the long-range, so-called electromagnetic enhancement of the RBD Raman spectrum to the short-range “chemical” enhancement via the reduction of the disulfide bonds and the interaction of the resultant thiol groups with the SERS-active surface. Further detailed study of the reduced RBD may shed more light on the nature of the SERS effect.

### 3.2. A New Method for SERS Response Enhancement by Applying an Additional Thin Metal Layer onto RBD Aggregates Deposited on the SERS-Active Substrate

The application of an additional, optically semitransparent silver layer (about 10 nm) onto protein aggregates formed by reduced RBD molecules led to a significant further enhancement of the SERS signal. Scheme 1 (right panel) illustrates the procedure of additional silver layer deposition, and Figure 5 (right column) shows the procedure for the spectral analysis of protein aggregates formed on the SERS-active substrate after additional silver coating. The images obtained in the wide-field upright optical microscopy mode (upper panels) and mapping (middle panels), based on the characteristic Raman band with a Stokes shift of 1344 cm^−1^ (C–H vibration) in the confocal mode, are shown. The essence of the procedure consisted in searching for RBD aggregates by means of a conventional microscope, obtaining a spectrum at a point, mapping aggregates in the confocal Raman microscopy mode (accumulation time, 0.4 s per point), and then obtaining a spectrum at the point of the highest intensity obtained during mapping.

**Figure 8:**
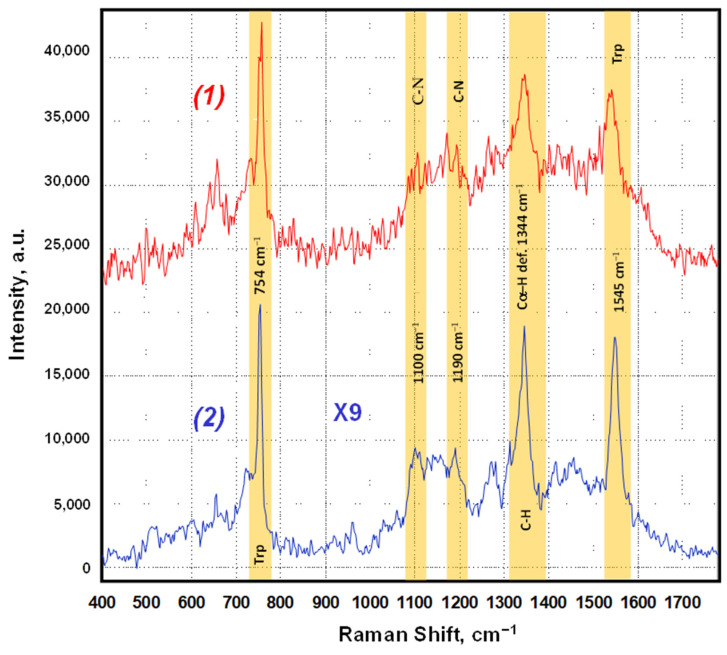
Changes in the degree of enhancement of the Raman scattering spectrum of a sample of the RBD of the SARS-CoV-2 S glycoprotein with reactive thiol groups upon application of a thin silver film onto the protein sample bound with the SERS-active substrate. (1) The SERS spectrum of the RBD upon additional application of a thin silver film onto the protein sample bound to the surface of the SERS-active substrate. The spectrum is not normalized. (2) The SERS spectrum of the RBD without additional application of a thin silver film onto the protein sample bound to the surface of the SERS-active substrate. The normalization factor for the tryptophan residue band at about 755 cm^−1^ is 9.0.

The SERS spectrum of a sample of reduced S glycoprotein RBD with reactive thiol groups bound to the SERS-active substrate recorded after the thin silver coating of the sample is shown in Figure 8. It can be seen that the deposition of an additional silver layer led to an almost tenfold enhancement of the SERS RBD signal compared to the SERS signal of the uncoated RBD. It is noteworthy that the same procedure of the deposition of an additional silver layer onto a film of the native RBD located on the same SERS-active substrate led to a complete disappearance of the Raman signal.

**Figure 9:**
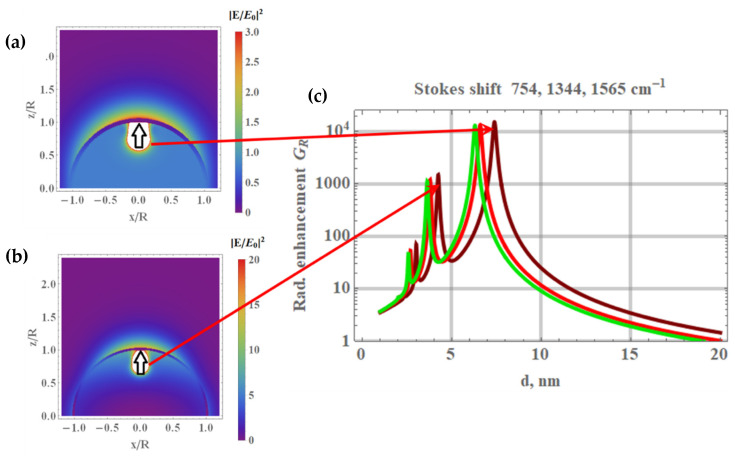
Radiation enhancement *G*_R_ of the Raman signal from the aggregate of molecules of RBD protein coated with a spherical silver envelope with a radius of *R* = 90 nm as a function of silver layer thickness d. (**a**,**b**) The electric field distribution in the dipole and quadrupole resonances excited by the molecular dipole, which is indicated with an open arrow; the dipole oscillates with a frequency of 2π*c*/(λ = 834 nm), which corresponds to the Trp Stokes shift (754 cm^−1^) from the fundamental laser frequency of 2π*c*/(λ = 785 nm); (**a**) the dipole resonance is excited in the silver envelop with a thickness of *d* = 8 nm; (**b**) the quadrupole resonance is excited at *d* = 4 nm; (**c**) radiation enhancement *G*_R_ (d); molecular dipoles are distributed over the internal surface of the silver envelope with a radius of *R* = 90 nm; the thickness of the dipole layer is 8 nm; the dipoles radiate at wavelengths of λ = 834, 878, and 895 nm (the brown, red, and green curves), which correspond to the main Stokes shifts (754, 1344, and 1565 cm^−1^) in the Raman signal from the RBD protein (cf. Figure 6 and Figure 7); the Raman signal from the plasmon resonator is normalized to the Raman signal generated by the bare molecular layer.

The additional enhancement of the SERS signal from the reduced RBD coated with an additional silver layer can be explained by an increase in the Q-factor of the metal–dielectric plasmon resonator. It is well known that, at optical frequencies, the permittivity of metals is negative. In our case, the conducting current flows in the silver shell of the resonator, and the polarization current oscillates in the dielectric RBD core. These currents have opposite directions because the metal permittivity is negative, and we obtain the plasmon resonances shown in Figure 9.

We have developed a simple model of SERS for molecules in a metal envelope, which is briefly presented below. The model qualitatively describes the enhancement of SERS from the RBD of the SARS-CoV-2 spike glycoprotein coated with a thin silver envelope. The theory estimates how the SERS changes when the RBD globule is covered with a metal nanolayer. The radiating molecular dipole, indicated by a black arrow in Figure 9a,b, interacts with the metal envelope and excites the surface plasmons. The plasmon radiation is either added to or subtracted from the molecular dipole radiation. The plasmon oscillations reach their maximum when the dipole frequency is close to the plasmon resonance of the metal envelope and the dipole itself is close to the plasmon envelope. The electric fields at the dipole and quadrupole resonances are shown in Figure 9a,b, respectively. The effective dipole of collective electronic vibrations may be much larger than the molecular dipole, which is itself enhanced due to the usual short-range SERS effect. Therefore, the radiation enhancement *G*_R_ is multiplied by the SERS enhancement factor *G*_2_, which is due to the direct interaction of the RBD molecular dipole with the metal surface. That is, the conventional SERS signal is multiplied by the factor *G*_R_ >> 1, shown in Figure 9c as a function of the thickness of the metal envelope. We can see that the overall radiation is considerably enhanced.

To estimate the radiation enhancement *G*_R_ due to the metal envelope, we use the simplest model, in which the radiating dipole is placed inside a hollow metal sphere with the radius *R* and with the spherical shell thickness *d*. The dipole radiation is partly reflected back by the sphere and partly goes outside the metal shell. The symmetry of the system allows the electromagnetic field **E**_i_ inside the metal sphere (*r* < *R*), the field **E**_m_ in the spherical shell (*R* < *r* < *R* + *d*), and the field **E**_e_ outside the sphere (*r* > *R* + *d*) to be expanded to Legendre polynomials *P*_n_ (cos *θ*). It is convenient to use a spherical coordinate system {*r*, *θ*, *ϕ*} and assume that the RBD molecular dipole is perpendicular to the surface of the metal envelope. This assumption corresponds to the orientation of the thiol bonds between the RBD protein and the metal surface. The direction of the RBD dipole is taken to be the *z* axis for the spherical coordinates. Then, the internal field **E**_i_ can be expanded to the following expression:

(1) $$\left\{E_{r},\;E_{\theta },\;E_{\varphi }\right\}=E_{dip}+\overset{\infty }{\underset{n=0}{\sum }}A_{n}\left(1+n\right){\left(r\;r_{0}\right)}^{n}\left\{\left(1+n\right)P_{1+n}\left(\cos\;\theta \right),\;P_{n+1}^{1}\left(\cos\;\theta \right),\;0\right\},$$

where **E***_dip_* is the dipole field, all distances are measured in terms of the radius *R* of the envelope, $P_{n+1}^{1}$ is the associated Legendre polynomial of degree *n* + 1 and first order, *r*_0_ < 1 is the distance to the dipole from the center of the envelope. To simplify the consideration, we neglect the retardation effects inside the sphere and in the metal. In other words, we use the quasistatic approximation to calculate the fields **E**_i_ and **E**_m_ for the values of the radius *r* < *R* + *d*. However, the exact solution of Maxwell’s equations is used to find the external fields **E**_e_ and **H**_e_, which extend to infinity. These fields **H**_e_ = **curl A** and **E**_e_ = *i* **curl H**_e_/*k* are calculated from the vector potential:

(2) $$A=\hat{z}\;k^{2}\sum_{n=0}^{\infty }D_{n}\;{\left(k\;r_{0}\right)}^{n}{\;z}_{0}^{n}\;h_{n}^{1}\left(kr\right)\;P_{n}\left(\cos\;\theta \right)/\left(2n-1\right)!!,$$

where $\hat{z}$ is the unit vector in the *z* direction, $h_{n}^{1}\left(kr\right)$ is the first-order Hankel function of the n-th degree, and *k* = *ω*/*c* is the wavevector.

The electric fields **E**_i_, **E**_m_, and **E**_e_ are found from the boundary conditions at the internal (*r* = *R*) and external (*r* = *R* + *d*) surfaces of the metal envelope. We have matched the tangential components of the electric fields and normal components of the electric displacement at the sphere boundaries. When we match the **E**_m_ and **E**_e_ fields at the external surface of the metal envelope, we assume the quasistatic limit *k* → 0 for the **E**_e_ field, expanding the Hankel function in Equation (2) in series of k. Thereby, we obtain, e.g., the expression for the coefficient *A*_n_ and *D*_n,_ Equations (1) and (2):

(3) $$A_{n}=\frac{\left(2+n\right)\left(\left(3+2n\right)\left(\varepsilon_{2}-\varepsilon_{d}\right)\varepsilon_{m}+t_{n}\left(\varepsilon_{m}-\varepsilon_{d}\right)\left(\left(2+n\right)\varepsilon_{2}+\left(1+n\right)\varepsilon_{m}\right)\right)}{DD},\;D_{n}=-\frac{{\left(3+2n\right)}^{2}\left(1+t_{n}\right)\varepsilon_{d}\varepsilon_{m}}{DD},$$

where t_n_ = (1 + *d*)^3+2*n*^ − *1*; *ε*_d_ ≈ 2, *ε*_m_, and *ε*_2_ = 1 are the permittivities of the internal space of the envelope filled with RBD protein, the metal (silver) envelope, and outer space (vacuum or air), respectively; the denominator *DD* in Equation (3) equals to

(4) $$\mathrm{DD}=\left(3+2n\right)\left(\left(2+n\right)\varepsilon_{2}+\left(1+n\right)\varepsilon_{d}\right)\varepsilon_{m}+t_{n}\left(\left(2+n\right)\varepsilon_{2}+\left(1+n\right)\varepsilon_{m}\right)\;\left(\left(1+n\right)\varepsilon_{d}+\left(2+n\right)\varepsilon_{m}\right).$$

Zeroing the determinant, we obtain the following dispersion equation determining the *n*th resonance:

(5) $${\left(1+\frac{d}{R}\right)}^{2n+3}=\frac{\left(1+n\right)\left(2+n\right)\left(\varepsilon_{d}-\varepsilon_{m}\right)\left(\varepsilon_{2}-\varepsilon_{m}\right)}{\left(\left(2+n\right)\varepsilon_{2}+\left(1+n\right)\varepsilon_{m}\right)\left(\left(1+n\right)\varepsilon_{d}+\left(2+n\right)\varepsilon_{m}\right)},$$

where *R* is the radius of the metal envelope, and the natural dimensions are restored. Equation (5) gives the resonance thickness *d* of the metal envelope for a given frequency and, hence, a fixed value of the metal permittivity *ε*_m_(*ω*) (see Figure 9). The metal permittivity *ε*_m_(*ω*) = *ε*_m1_(*ω*) + *iε*_m2_(*ω*) has a small imaginary part *ε*_m2_(*ω*)/|*ε*_m1_(*ω*)| << 1. It is noteworthy that the permittivity is mostly negative in the optical and infrared spectral ranges in “good” optical metals, such as silver and gold. The resonant frequencies of the studied system ω_n_ are determined from Equation (5), where *ε*_m_(*ω*) is replaced by its real part *ε*_m1_(*ω*) and the numbers *n* correspond to the resonance conditions of excitation of the dipole, quadrupole, hexapole, etc., resonances. The field in the silver sphere is considerably enhanced in the case of plasmon resonances. It can exceed the field of the bare dipole. Moreover, the effective dipole moment of a plasmonic resonator can exceed the bare molecular dipole moment by orders of magnitude (see Figure 9c). When the external electric (**E**_e_) and magnetic (**H**_e_) fields are known, we can find the energy flux out of the plasmonic sphere (Poynting vector) and compare it with the flux from a bare dipole. Thus, we find the radiation enhancement factor,

(6) $$G_{R}=\overset{\infty }{\underset{n=0}{\sum }}\frac{3{\left(3+2n\right)}^{3}\left(n^{2}+n-1\right){\left(1+t_{n}\right)}^{2}{\left(kr_{0}\right)}^{2n}}{\left(4n^{2}-1\right){\left(\left(2n-1\right)!!\right)}^{2}}\;{\left[\frac{\varepsilon_{d}\varepsilon_{m}}{DD}\right]}^{2},$$

for a dipole placed at the distance *r_0_* from the center of the metal sphere; the determinant *DD* is given by Equation (4). Raman scattering is noncoherent; therefore, it is enough to average the enhancement factor *G*_R_ over the dipoles.

In a typical Raman experiment, the excitation laser frequency *ω*_0_ is determined by the experimental setup; therefore, the frequencies *ω* = *ω*_0_ − *ω*_s_ of the molecular dipoles are also fixed. On the other hand, the thickness *d* of the metal envelope is rather easily changed during sample preparation. From Equation (5), it can be concluded that there is a resonant metal thickness for each dipole frequency *ω*. In our experiment, the laser wavelength was 785 nm. Using this value, we calculated the factor of radiation enhancement due to the metal envelope (*G*_R_) as a function of the thickness *d* for the fixed wavelengths of 832, 878, and 895 nm, which correspond to the most prominent Trp spectral lines, with Stokes shifts of 754 and 1565 cm^−1^ and the C–H bond vibration line at 1344 cm^−1^, respectively (see Figure 6 and Figure 7). The radius of the silver envelope *R* = 90 nm was chosen so that its volume 4π*R*^3^/3 was equal to the typical volume *V*_ef_ of the reduced RBD aggregate (~10^6^ nm^3^) estimated above. The plot of the radiation enhancement factor *G*_R_ as a function of the envelope thickness *d* is shown in Figure 9. We obtain the growth of the enhancement factor *G*_R_ by an order of magnitude for the metal thickness of *d*~10 nm, which corresponds to the experimental values. Note that the Stokes lines with shorter wavelengths are enhanced more than those with longer wavelengths, which also agrees well with the experimental spectrum in Figure 7. It is supposed that the radiating dipoles are distributed in a thin layer at the inner surface of the sphere. The layer thickness is about 8 nm, which corresponds to a monolayer of reduced RBD molecules.

As can be seen from Figure 9, the enhancement factor *G*_R_, in general, increases as the thickness *d* of the metal envelope is decreased below 10 nm. On the other hand, the metal film becomes semicontinuous when its thickness is decreased. Thus, the optimal parameters of the proposed “envelope” method for SERS signal enhancement are the subject for further consideration.

It is natural to think that the electric field enhancement results in the enhancement of the dipole radiation. Usually, the dipole approximation is used to simulate the radiation of nanoparticles, because their size is much smaller than the wavelength. However, if the dipole interacts with an optical nearfield, it experiences strong perturbations caused by field gradients. This increases the contribution of the multipoles and can lead to alteration of the selection rules. In summary, our simple spherical model qualitatively describes the enhancement of SERS from the RBD of the spike protein when its aggregate is coated with a thin silver film. Moreover, our results offer the opportunity to design effective SERS sensors, where the initial SERS signal is further enhanced by the factor *G*_R_ >> 1.

## 4. Conclusions

The main condition for the efficiency of the SERS method as applied to specific detection and diagnosis is the formation of Raman-enhancing surfaces or cavities capable of concentrating incident light within small volumes containing the analyte. The present study is the first to succeed in label-free recording of protein-specific SERS spectra at the analyte concentrations sufficiently low for detecting the viral protein antigen at physiologically relevant (sub-femtogram) levels. For this purpose, we employed concentration of light in a metal–dielectric microcavity formed via the interaction between the thiol groups of the reduced RBD of SARS-CoV-2 S glycoprotein and a silver surface. Furthermore, a plasmonic nanoresonator was formed by coating the dielectric RBD aggregates with nanometer-thick silver envelopes. The nanoresonator provides an additional tenfold enhancement of the SERS signal. Moreover, the SERS signal of the reduced RBD is not only a hundred times more intense than that of the native RBD, but also has a completely different shape, dominated by the signal of Trp amino acid residues. In the native RBD, they are buried in the protein interior and do not provide any signals in either the Raman or the SERS spectra. It has been shown that the formation of chemical bonds between the thiol groups of the reduced RBD and the surface of the SERS-active substrate activates a short-range “chemical” mechanism of Raman signal enhancement. To the best of our knowledge, this is the first report on the direct transition from the long-range electromagnetic enhancement of the RBD Raman spectrum to its short-range “chemical” enhancement. This is caused by the specific reduction of the protein disulfide bonds and the resultant direct interaction of not only the thiol groups, but also the aromatic Trp groups with the surface of the SERS-active substrate. We speculate that the combination of the thiol bond reduction/implantation with the conversion of a molecular aggregate into a plasmon nanoresonator can be developed into a new method of SERS analysis of various proteins and other large organic molecules.

Importantly, all the studies on the SERS detection of SARS-CoV-2 viral particles or its protein antigens published to date are indirect. They record the SERS spectra of the so-called SERS nanotags labeled with low-molecular-weight Raman reporters, or those of the receptors or aptamers binding S glycoprotein, rather than the SERS spectra of the viral antigens themselves. The use of such indirect approaches makes the studies of the viral protein antigen structures and their differences in different viral variants impossible. Our study is the first to obtain typical and characteristic Raman and SERS spectra of the RBD of S glycoprotein, the key SARS-CoV-2 viral antigen. Here, we have demonstrated the possibility of label-free detection of the characteristic spectra of viral protein antigens at concentrations orders of magnitude lower than those required for the detection of the whole virus in biological media without using multicomponent analysis. This makes the development of a high-performance optical method for the detection of pathogen variants and their conformation analysis a highly realistic task.

## Data Availability

The datasets generated during and/or analyzed during the current study are available from the corresponding author on reasonable request.

## Supplementary Materials

The following supporting information [27,37,38,39,40,41] can be downloaded at https://www.mdpi.com/article/10.3390/bios12050300/s1, Supplementary Tables: Supplementary Table S1: spike glycoprotein RBD amino acid sequence, Supplementary Table S2: physico-chemical properties of RBD protein, Supplementary Figure S1: scanning electron microscopy photograph.
