# Supplementary material for: Label-Free Detection of the Receptor-Binding Domain of the SARS-CoV-2 Spike Glycoprotein at Physiologically Relevant Concentrations Using Surface-Enhanced Raman Spectroscopy
Source: Biosensors (Basel). 2022 May 5;12(5):300. doi: 10.3390/bios12050300 (PMC9138710; doi:10.3390/bios12050300)
Supplement: Supplementary file 1 [file biosensors-12-00300-s001.zip › biosensors-1690418-supplementary.pdf]

biosensors

MDPI

Supplementary Materials

Label-Free Detection of the Receptor-Binding Domain of the SARS-CoV-2 Spike Glycoprotein at Physiologically Relevant Concentrations Using Surface-Enhanced Raman Spectroscopy

Andrey K. Sarychev [1], Alyona Sukhanova [2], Andrey V. Ivanov [1], Igor V. Bykov [1], Nikita V. Bakholdin [3], Daria V. Vasina [4], Vladimir A. Gushchin [4,5], Artem P. Tkachuk [4], Galina Nifontova [2,6], Pavel S. Samokhvalov [6], Alexander Karaulov [7] and Igor Nabiev [2,6,7,*]

[1] Institute of Theoretical and Applied Electrodynamics, Russian Academy of Sciences, 125412 Moscow, Russia; sarychev_andrey@yahoo.com (A.K.S.); av.ivanov@physics.msu.ru (A.V.I.); bykov.i.v@yandex.ru (I.V.B.)
[2] Laboratoire de Recherche en Nanosciences, LRN-EA4682, Université de Reims Champagne-Ardenne, 51100 Reims, France; alyona.sukhanova@univ-reims.fr(A.S.); galina.nifontova@univ-reims.fr (G.N.)
[3] Moscow Power Engineering Institute, National Research University, 111250 Moscow, Russia; baholdin.n@mail.ru
[4] Gamaleya National Research Centre for Epidemiology and Microbiology, Ministry of Health of the Russian Federation, 123098 Moscow, Russia; d.v.vasina@gmail.com (D.V.V.); vladimir.a.gushchin@gamaleya.org (V.A.G.); artem.p.tkachuk@gamaleya.org (A.P.T.)
[5] Department of Virology, Biological Faculty, Lomonosov Moscow State University, 119234 Moscow, Russia
[6] Moscow Engineering Physics Institute, National Research Nuclear University MEPhI, 115409 Moscow, Russia; p.samokhvalov@gmail.com (P.S.S.)
[7] Department of Clinical Immunology and Allergology, Institute of Molecular Medicine, Sechenov First Moscow State Medical University (Sechenov University), 119146 Moscow, Russia; drkaraulov@mail.ru
* Correspondence: igor.nabiev@univ-reims.fr

Supplementary Table S1. Spike glycoprotein RBD amino acid sequence.
The RBD includes 223 amino acid residues, from Arg319 (R319) to Phe541 (F541) of the spike glycoprotein amino acid sequence.
The aromatic amino acid residues and the cysteine residues are marked by colors.

319 329 339 349
RVQPTESIVR FPNITNLC1PF GEVFNATRFA SVYAWNRKRI [1]
359 369 379 389
SNC1VADYSVLYNSASFSTFKC2YGVSPTKLN DLC3FTNVYAD
399 409 419 429
SFVIRGDEVR QIAPGQTGKI ADYNYKLPDD FTGC2VIAWNS
439 449 459 469
NNLDSKVGGN YNYLYRLFRK SNLKPFERDI STEIYQAGST
479 489 499 509
PC4NGVEGFNC4 YFPLQSYGFQ PTNGVGYQPY RVVVLSFELL
519 529 539
HAPATVC3GPK KSTNLVKNKC VNF

[1] The color codes of the marked aromatic amino acid and cysteine residues:
F: Phenylalanine Phe
Y: Tyrosine Tyr
W: Tryptophan Trp
C: Cysteine Cys
C1, C2, C3 and C4 are the cysteine residues forming four disulfide bonds within the three-dimensional RBD structure.

Supplementary Table S2. Physico-chemical properties of RBD protein.
RBD molecular weight: 25 098.40 Da
RBD theoretical pI: 8.91
RBD amino acid composition (223 amino acid residues):

Ala	(A)	12	5.4%
Arg	(R)	11	4.9%
Asn	(N)	21	9.4%
Asp	(D)	9	4.0%
**Cys**	**(C)**	**9**	**4.0%**
Gln	(Q)	7	3.1%
Glu	(E)	7	3.1%
Gly	(G)	15	6.7%
His	(H)	1	0.4%
Ile	(I)	9	4.0%
Leu	(L)	14	6.3%
Lys	(K)	12	5.4%
Met	(M)	0	0.0%
**Phe**	**(F)**	**16**	**7.2%**
Pro	(P)	13	5.8%
Ser	(S)	17	7.6%
Thr	(T)	13	5.8%
**Trp**	**(W)**	**2**	**0.9%**
**Tyr**	**(Y)**	**15**	**6.7%**
Val	(V)	20	9.0%

Supplementary Figure S1. Scanning Electron Microscopy Photograph

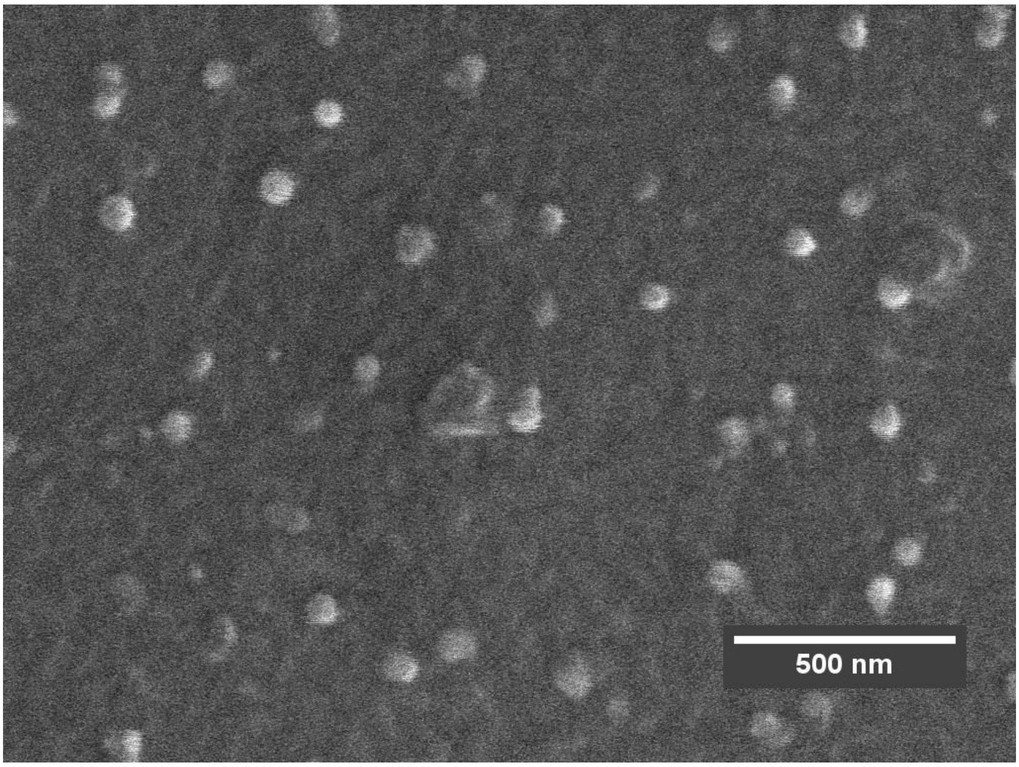

SEM measurements were carried out by using a scanning electron microscope with variable pressure ZEISS/LEO 1455vp (Germany).

Supplementary References

1. Zavyalova, E.; Ambartsumyan, O.; Zhdanov, G.; Gribanyov, D.; Gushchin, V.; Tkachuk, A.; Rudakova, E.; Nikiforova, M.; Kuznetsova, N.; Popova, L.; et, al. SERS-Based Aptasensor for Rapid Quantitative Detection of SARS-CoV-2. *Nanomaterials* **2021**, 11, 1394.
2. Peng, Y.; Lin, C.; Long, L.; Masaki, T.; Tang, M.; Yang, L.; Liu, J.; Huang, Z.; Li, Z.; Luo, X., et, al. Charge-Transfer Resonance and Electromagnetic Enhancement Synergistically Enabling MXenes with Excellent SERS Sensitivity for SARS-CoV-2 S Protein Detection. *Nano micro Lett.* **2021**, *13*, 1–7.
3. Chen, H.; Park, S.G.; Choi, N.; Kwon, H.J.; Kang, T.; Lee, M.K.; Choo, J. Sensitive Detection of SARS-CoV-2 Using a SERS-Based Aptasensor. *ACS sens.* **2021**, *6*, 2378–2385.
4. Yang, Y.; Peng, Y.; Lin, C.; Long, L.; Hu, J.; He, J.; Zeng, H.; Huang, Z.; Li, Z.Y.; Tanemura, M., et, al. Human ACE2-functionalized gold "virus-trap" nanostructures for accurate capture of SARS-CoV-2 and single-virus SERS detection. *Nano micro Lett.* **2021**, *13*, 1–13.
5. Huang, G.; Zhao, H.; Li, P.; Liu, J.; Chen, S.; Ge, M.; Qin, M.; Zhou, G.; Wang, Y.; Li, S., et, al. Construction of Optimal SERS Hotspots Based on Capturing the Spike Receptor-Binding Domain (RBD) of SARS-CoV-2 for Highly Sensitive and Specific Detection by a Fish Model. *Anal. Chem.* **2021**, *93*, 16086–16095.
6. Akdeniz, M.; Ciloglu, F.U.; Tunc, C.U.; Yilmaz, U.; Kanarya, D.; Atalay, P.; Aydin, O. Investigation of mammalian cells expressing SARS-CoV-2 proteins by surface-enhanced Raman scattering and multivariate analysis. *Analyst* **2022**, *147*, 1213-1221.
